# Production and Stabilization of Specific Upregulated Long Noncoding RNA HOXD-AS2 in Glioblastomas Are Mediated by TFE3 and miR-661, Respectively

**DOI:** 10.3390/ijms23052828

**Published:** 2022-03-04

**Authors:** Yiming Qin, Yingjiao Qi, Xin Zhang, Zhiang Guan, Wei Han, Xiaozhong Peng

**Affiliations:** State Key Laboratory of Medical Molecular Biology, Medical Primate Research Center, Neuroscience Center, Department of Molecular Biology and Biochemistry, Institute of Basic Medical Sciences, Chinese Academy of Medical Sciences, School of Basic Medicine of Peking Union Medical College, Beijing 100005, China; oranmigi@163.com (Y.Q.); ying7jiao@126.com (Y.Q.); zx18004028155@163.com (X.Z.); Guanzhiang2021@163.com (Z.G.)

**Keywords:** glioma, *HOXD-AS2*, *TFE3*, *miR-661*

## Abstract

Differential expression of long noncoding RNAs (lncRNA) plays a key role in the development of gliomas. Because gliomas are the most common primary central nervous system tumor and glioblastomas have poor prognosis, it is urgent to develop new diagnostic methods. We have previously reported that lncRNA *HOXD-AS2*, which is specifically up-regulated in gliomas, can activate cell cycle and promote the development of gliomas. It is expected to be a new marker for molecular diagnosis of gliomas, but little is known about *HOXD-AS2*. Here, we demonstrate that *TFE3* and *miR-661* maintain the high expression level of *HOXD-AS2* by regulating its production and degradation. We found that *TFE3* acted as a transcription factor binding to the *HOXD-AS2* promoter region and raised H3K27ac to activate *HOXD-AS2*. As the cytoplasmic-located lncRNA, *HOXD-AS2* could be degraded by *miR-661*. This process was inhibited in gliomas due to the low expression of *miR-661*. Our study explains why *HOXD-AS2* was specifically up-regulated in gliomas, helps to understand the molecular characteristics of gliomas, and provids insights for the search for specific markers in gliomas.

## 1. Introduction

Glioma accounts for nearly 80% of primary tumors in the central nervous system. Glioblastoma multiforme (GBM, glioblastoma) is the most aggressive, invasive tumor of gliomas [1,2]. Patients with glioblastomas have a poor prognosis, and only have a mean survival of 14.6 months among adults [3,4]. However, some molecular features, such as isocitrate dehydrogenase 1 (*IDH-1*) mutation, confer a favorable prognosis [5]. *IDH* wild-type glioblastomas typically contain higher rates of epidermal growth factor receptor gene (*EGFR*) amplification and phosphatase and tensin homolog (*PTEN*) deletions [5,6,7]. O6-methylguanine-DNA methyltransferase (*MGMT*) promoter methylation is initially identified as a prognostic and predictive marker within the diagnosis of GBM in patients treated with temozolomide [8]. *IDH* mutation is thought to be followed by other mutations, such as *TP53*, *ATRX* (astrocytoma) or co-deletion of 1p/19q (oligodendroglioma) [9,10]. To improve the glioma treatment, it is essential to get a better understanding of the molecular information of gliomas and develop new therapeutic strategies.

Long noncoding RNAs (lncRNA) are defined as a class of noncoding RNAs of more than 200 nucleotides (nt) in length [11]. Recent studies have demonstrated that the expression of lncRNAs is altered in tumors, and these changes can serve as markers for cancer diagnosis and potential drug targets [12]. LncRNAs are involved in cancer-related signaling pathways, affecting cell proliferation, angiogenesis, cell cycle, tumor invasion and a series of biological processes related to cancer genesis and development [13,14,15].

Some lncRNAs are highly expressed in gliomas, which have been reported to promote cell proliferation, migration, and invasion. The roles played by lncRNAs in tumors deserve in-depth study. We will have a more comprehensive understanding of lncRNAs and their corresponding molecular mechanisms that are involved in various key stages of cancer development and progression. On this basis, lncRNAs are expected to become new targets for tumor therapy [16].

We previously found that lncRNA HOXD Cluster Antisense RNA 2 (*HOXD-AS2*) was up-regulated in glioma cells by lncRNA microarray. Then, we confirmed that *HOXD-AS2* promoted the proliferation of glioblastoma cells and the cell cycle progression; we also demonstrated that the growth of tumors was significantly constrained when silencing *HOXD-AS2* in vivo [17]. It has also been found that the activation of the transforming growth factor beta (TGF-β) signaling pathway can up-regulate *HOXD-AS2* in high-expressed *MGMT* glioma cells [18]. *HOXD-AS2* can affect the invasion and apoptosis of gliomas by targeting miR-3681-5p and activating the metastasis associated in lung adenocarcinoma Transcript 1 (*MALT1*) through competing endogenous RNA (ceRNA) mechanism [19]. Moreover, *HOXD-AS2* inhibits the PI3K/Akt signaling pathway by targeting the neighboring gene *HOXD8* and acts as a tumor suppressor gene in gastric cancer [20]. Overall, there are few studies on *HOXD-AS2*, particularly the upstream mechanism of *HOXD-AS2*.

Interestingly, the pan-cancer analysis of *HOXD-AS2* demonstrated that the overexpression was specific in gliomas. To explore the reason of this phenomenon, we analyzed the upstream mechanism of *HOXD-AS2* in gliomas. We demonstrated that the transcription factor binding to IGHM enhancer 3 (*TFE3*) and *hsa-miR-661* (*miR-661*) regulated *HOXD-AS2* transcriptional activity and transcript stability. *TFE3*, located on chromosome Xp11.23, encodes a transcription factor containing the Basic Helix-Loop-Helix (BHLH) domain that binds to the MUE3 type E box sequence in the gene promoter, which coding protein promotes the expression of genes downstream of the TGF-β signal [21,22,23]. Fusion of *TFE3* and several different partner genes occurs in several types of tumors, results in nuclear expression of the *TFE3* protein. The main tumors associated with *TFE3* gene fusion are renal cell carcinoma, alveolar soft tissue sarcoma, partial epithelioid hemangioendothelioma (EHE), some perivascular epithelioid cell tumors and rare ossifying fibromyxoid tumors [24,25,26]. *miR-661* is frequently reported as a down-regulated gene in gliomas, which inhibits the proliferation, migration and invasion of glioma cells by targeting *hTERT*, and participates in ceRNA regulation [27,28].

Our studies reveal the molecular mechanism explaining why *HOXD-AS2* was specifically overexpressed in gliomas and provide a theoretical basis for the future of *HOXD-AS2* as a potential molecular diagnostic marker.

## 2. Results

### 2.1. HOXD-AS2 Is Up-Regulated Specifically in Gliomas and Acts as a Risk Factor

**Figure 1:**
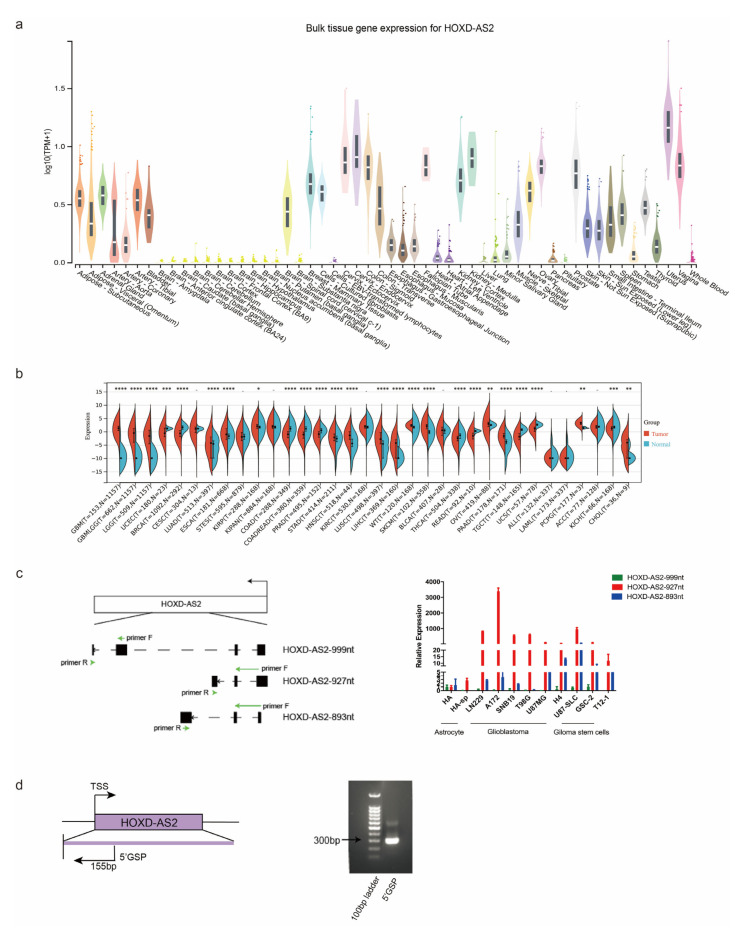

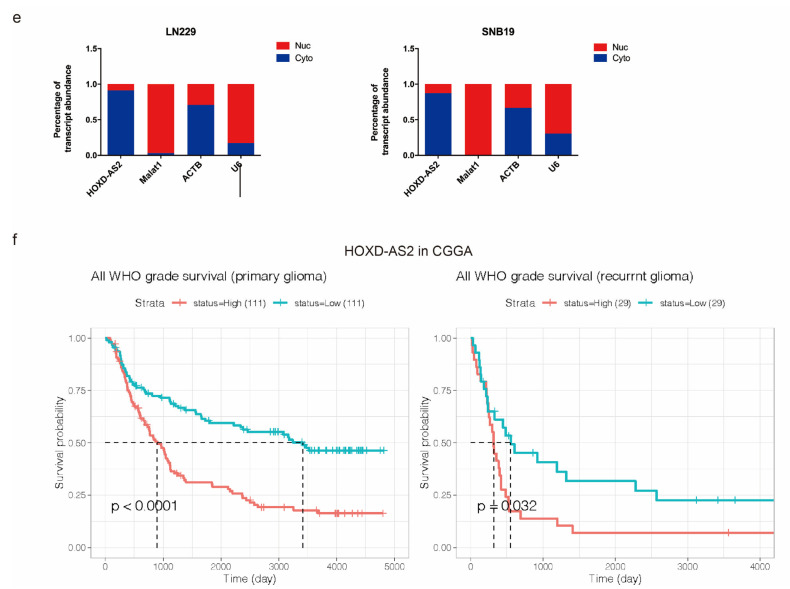
*HOXD-AS2* is up-regulated in gliomas and correlated with prognosis. (**a**) UCSC database showed the expression of *HOXD-AS2* in normal tissues (http://genome.ucsc.edu, accessed on 23 January 2022). (**b**) The expression of *HOXD-AS2* in different cancer types analyzed by TCGA and GTEx database. The *p*-values were calculated using a Wilcoxon test by R (version 4.0.5). Significant results were presented as * *p* < 0.05, ** *p* < 0.01, *** *p* < 0.001, **** *p* < 0.0001. (**c**) Pattern diagram of the specific primers for *HOXD-AS2* three transcripts (left). Relative expression of *HOXD-AS2* in astrocyte cell lines, glioblastoma cell lines and glioma stem cell lines were quantified by RT-qPCR (right). (**d**) *HOXD-AS2* full-length identification by 5′RACE. (**e**) Detecting the expression of *HOXD-AS2* in cytoplasm and nucleus by RT-qPCR. *MALAT1*, *ACTB* and *U6* as positive controls. (**f**) Kaplan–Meier survival analysis of OS in GBM patients based on *HOXD-AS2* expression in CGGA (Mantel–Cox test).

We analyzed the expression level of *HOXD-AS2* in different kinds of normal tissues via UCSC database. The data demonstrated that *HOXD-AS2* was expressed at a low level in brain tissues (Figure 1a). Then, we used The Cancer Genome Atlas (TCGA) [29] and The Genotype-Tissue Expression (GTEx) [30] data and found that *HOXD-AS2* was specifically up-regulated in glioblastomas and low-grade gliomas (LGG) compared to other cancers (Figure 1b). This phenomenon suggested that *HOXD-AS2* might play an important role in the development of gliomas. According to the UCSC database, three transcripts of *HOXD-AS2* had been reported. We designed three transcripts’ specific primers. RT-qPCR showed that the transcript *HOXD-AS2*-927nt (nucleotides) was significantly up-regulated in six glioblastoma cell lines (LN229, A172, SNB19, T98G, U87MG and H4) compared with the astrocyte cell lines (HA and HA-sp) (Figure 1c). The result demonstrated that the expression of *HOXD-AS2* was high in LN229 and A172; thus, we mainly chose these two glioblastoma cell lines for the subsequent study. U87MG, as a more common glioblastoma cell line, would also be used. Next, we identified the full length of *HOXD-AS2* by 5′rapid amplification of cDNA ends (5′RACE). We used the gene-specific primer (GSP) for PCR, then, the sequencing result showed that 60 nucleotides at 5′end of *HOXD-AS2* were extended compared with the UCSC database (Figure 1d). To ascertain the localization of *HOXD-AS2* in glioma cells, RT-qPCR showed that *HOXD-AS2* was a kind of mainly cytoplasmic-localized lncRNAs (Figure 1e). In addition, we analyzed the expression of *HOXD-AS2* in different progression status, subtypes and IDH mutation status in the Chinese Glioma Genome Atlas (CGGA) cohort [31] (Figure S1a–c), finding no significant differences. But the expression of *HOXD-AS2* was lower in gliomas with 1p19q co-deletion [32] (Figure S1d). Next, we investigated the relationship between *HOXD-AS2* expression and glioma prognosis. Survival analysis using the Kaplan–Meier method revealed that high-expression *HOXD-AS2* group (*n* = 153) had a shorter overall survival (OS) in glioma patients than the low-expression group (*n* = 157) (Figure 1f). Overall, these results indicated that *HOXD-AS2* upregulation might play a critical role in the development and progression of gliomas. 

### 2.2. HOXD-AS2 Has No Abnormal DNA Copy Numbers in Gliomas and Its Expression Is Activated at the Transcriptional Level

**Figure 2:**
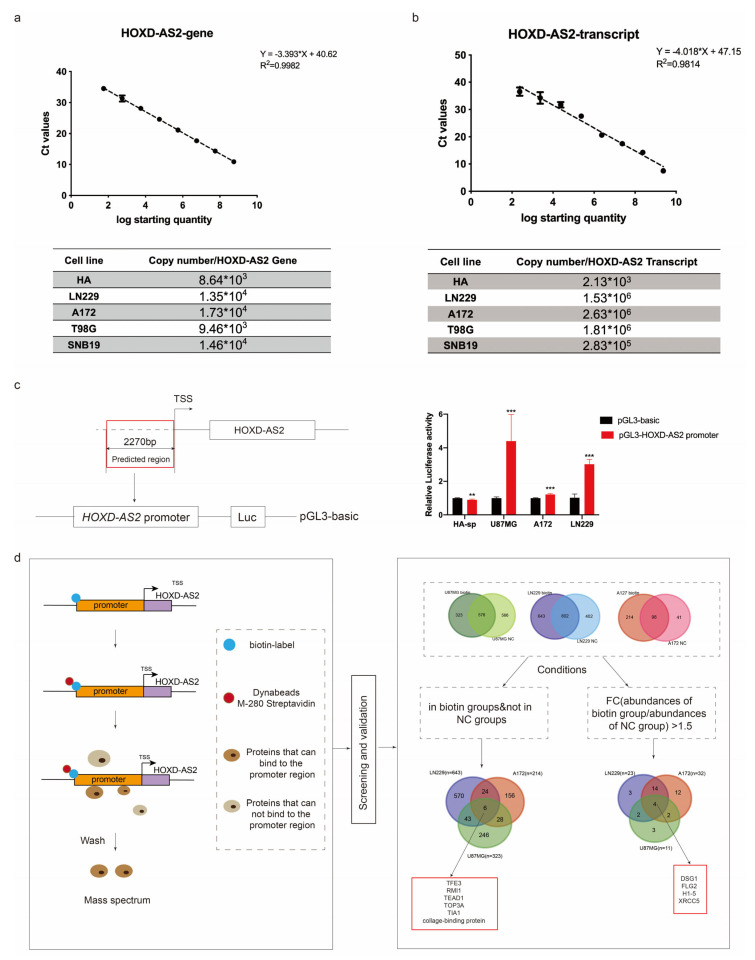

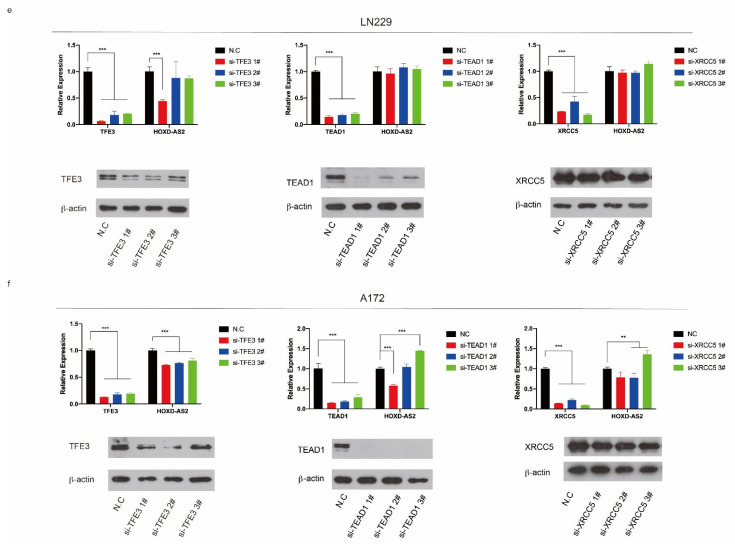
Overexpression of *HOXD-AS2* in glioblastomas was associated with transcriptional activation. (**a**,**b**) Absolute quantitative PCR in gene level (**a**) and transcription level (**b**). (**c**) The promoter region of *HOXD-AS2* transcriptional activity was detected by luciferase report assay in astrocyte cell HA-sp and glioblastoma cell lines LN229, A172 and U87MG. (**d**) DNA-pull down/MS assay was used to find the factors which binding to the *HOXD-AS2* promoter. Flow chart provided for screening of potential transcription factors regulating *HOXD-AS2*. (**e**,**f**) The expression of *HOXD-AS2* was detected by RT-qPCR and Western blot after transfecting with *TFE3*/*TEAD1*/*XRCC5* siRNAs in LN229 (**e**) and A172 (**f**). Data are presented as the mean ± SEM from three independent experiments. Significant results were presented as ** *p* < 0.01, *** *p* < 0.001. Two-tailed Student’s *t*-test was used in (**d**). (**e**,**f**) were analyzed by ANOVA.

As we all know, loss of heterozygosity (LOH) is the molecular genetics feature of oligodendrocyte. *EGFR* amplification and exon 2–7 deletion are the most common *EGFR* mutations in high-grade gliomas. Patients with *EGFR* amplification mutations have a poor prognosis [33]. Abnormal amplification or deletion of chromosomes is common in cancers. To test whether the high expression of *HOXD-AS2* in gliomas is caused by copy number variations (CNVs), we observed CNVs at the *HOXD-AS2* locus by the database cBioportal [34,35]. Compared with *EGFR*, as an amplification with a high incidence in gliomas, there was no significant copy number amplification at the *HOXD-AS2* locus (Figure S1e). Then, we used absolute quantitative PCR to detect *HOXD-AS2* expression in the astrocyte cell HA and glioblastoma cell lines, including LN229, A172, T98G and SNB19, at the level of gene and transcript. *HOXD-AS2* did not significantly amplify in glioblastoma cells at the level of gene but was up-regulated at the transcription level (Figure 2a,b), suggesting that the upregulation of *HOXD-AS2* was not associated with the abnormal amplification.

We thus presumed whether *HOXD-AS2* is aberrantly transcriptionally activated in glioblastomas. According to the UCSC database, we selected a sequence with the length of 2270 nucleotides upstream of *HOXD-AS2* transcription start site and inserted it into the pGL3-basic plasmid to detect the relative luciferase activity. Higher reporter gene activity was detected in glioblastoma cell lines, including LN229, A172 and U87MG, than the astrocyte cell HA-sp. The pGL3-basic empty vector was used as the negative control (Figure 2c). Results indicated that the transcriptional activity of the upstream of *HOXD-AS2* transcription start site was enhanced.

Then, we used JASPAR [36], ChIPBase [37] and promo (http://alggen.lsi.upc.es/cgi-bin/promo_v3/promo/promoinit.cgi?dirDB=TF_8.3, accessed on 23 January 2022) to search transcription factors binding sites in *HOXD-AS2* promoter, with 3000 nucleotides upstream of *HOXD-AS2* transcription start site used as the sequence for prediction. Based on the results, we analyzed the correction of *HOXD-AS2* and transcription factors in the TCGA and CGGA cohort. Supplementary Table S1 provided for all predicted genes. First, HOXD13 and HOXC9, which had the strongest correlation with *HOXD-AS2*, were selected to verify whether knockdown of *HOXD13* or *HOXC9* could down-regulate *HOXD-AS2*. RT-qPCR showed the expression of *HOXD-AS2* was not decreased (Figure S2a). Then, we used the same method to verify *USF1*, *E2F1* and *EBF1*, which got the highest score in all genes, but the expression of *HOXD-AS2* could not be down-regulated by knocking down any one of them (Figure S2b). According to the previous reports, the expression of *HOXD-AS2* is up-regulated through the activation of TGF-β signaling pathway in high-expression *MGMT* gliomas [18]. We analyzed the expression of *MGMT* in seven glioblastoma cell lines. The treatment of non-drug-resistant glioblastoma cell lines (LN229 and A172) with TGF-β1 did not affect the expression of *HOXD-AS2* (Figure S2c), and knocking down *SMAD2*, a key transcription factor in TGF-β signaling pathway, also did not inhibit the expression of *HOXD-AS2* (Figure S2d). These results suggested that there had other mechanisms regulating *HOXD-AS2* expression in gliomas with *MGMT* low expression. Therefore, we chose glioblastoma cell lines with low expression of *MGMT* to explore the reason why *HOXD-AS2* expression was up-regulated instead of T98G, a glioblastoma cell line with high expression of *MGMT*. It has been proven that *STAT3* can regulate the expression of *HOXD-AS1* in hepatocellular carcinoma [38]. *HOXD-AS1* is located upstream of *HOXD-AS2*. We found that upstream of *HOXD-AS2* transcription start site also had *STAT3* binding sites by using JASPAR analysis. However, the expression of *HOXD-AS2* could not be reduced by knocking down *STAT3* (Figure S2e).

To identify the potential transcription factors that interacted with the upstream of *HOXD-AS2*, we amplified the 5′ biotin-labeled *HOXD-AS2* promoter region located 2270 base pairs (bp) upstream of *HOXD-AS2* and used the unlabeled sequence as the negative control (NC). The 5′ biotin-labeled *HOXD-AS2* promoter was subsequently used to pull-down its associated transcription factors in three glioblastoma cell lines (LN229, A172 and U87MG). After mass spectrometric (MS) detection, all the proteins obtained would be taken to intersect in three glioma cells. In total, six proteins were found only in all biotin groups and not in NC groups, suggesting that these proteins were specifically pulled down by biotin-labeled *HOXD-AS2* promoter region. For further screening, we indicated four proteins, which expressed in biotin groups higher than in NC groups (biotin group/NC group, fold change and FC > 1.5) (Figure 2d). 

Before that, we analyzed ChIP-seq data of the glioblastoma cell U87MG and astrocyte cells from Cistrome database [39]. The database showed that higher levels of H3K4me3 and H3K27ac were found in the upstream of *HOXD-AS2* in U87MG (Figure S2f). Of those ten proteins, we screened transcription factor binding to IGHM enhancer 3 (*TFE3*), TEA domain transcription factor 1 (*TEAD1*) and X-ray repair cross complementing 5 (*XRCC5*), as candidate transcription factors that could bind to the *HOXD-AS2* promoter, which had been reported to participate in histone modifications. 

*TFE3* can act as an activated regulator and promote the assembly of an active enhancer repertoire, as evidenced by the deposition of H3K27ac, which then rapidly sequesters cohesion at inflammatory response enhancer-gene promoter clusters [40]. In colorectal cancer, *KDM3A* depletion causes H3K9me2 up-regulation mainly on TEAD1-binding enhancers rather than gene bodies, further resulting in H3K27ac decrease, less *TEAD1* binding on enhancers and transcription impair [41]. H3K27ac is an important marker of activity enhancer with E1A-binding protein p300 and CREB-binding protein (CREBBP/CBP) as catalysts [42]. p300 interacts with *XRCC5* by its HAT acetylating *XRCC5* in colon cancers and cooperates with *XRCC5* to regulate *COX-2* expression through acetylating *XRCC5* to promote colon cancer growth in vitro and in vivo [43].

It was found that only *TFE3* could stably regulate the expression of *HOXD-AS2* in two glioblastoma cell lines, LN229 and A172 (Figure 2e,f). Therefore, we hypothesized that *TFE3* was the transcription regulator upstream of *HOXD-AS2* in glioblastomas.

### 2.3. TFE3 Arises H3K27ac in Glioblastoma Cells and Accelerates the Expression of HOXD-AS2

**Figure 3:**
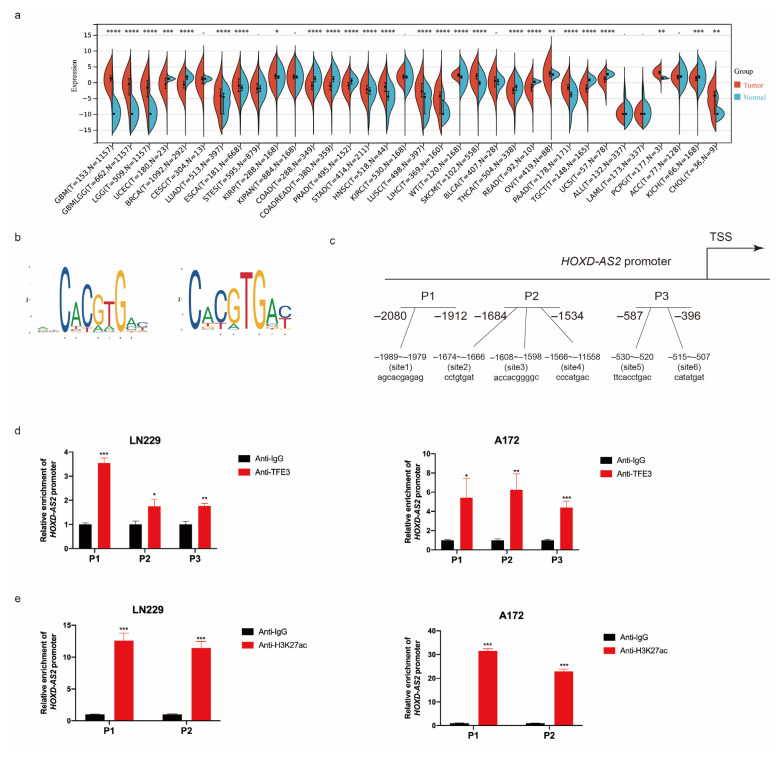

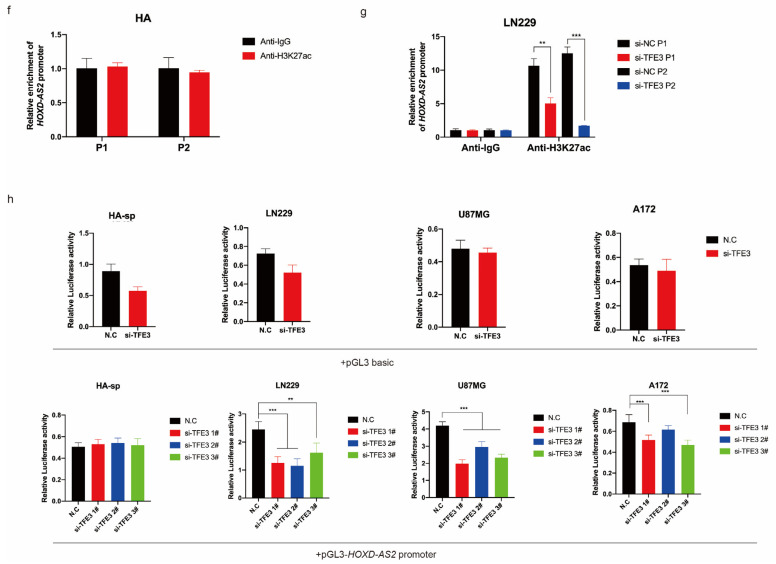
*TFE3* regulated the expression of *HOXD-AS2*. (**a**) TCGA and GTEx database demonstrated the expression of *TFE3* in different tumors. The *p*-values were calculated using a Wilcoxon test by R (version 4.0.5). Significant results were presented as * *p* < 0.05, ** *p* < 0.01, *** *p* < 0.001, **** *p* < 0.0001. (**b**,**c**) The binding motifs of *TFE3* (**b**) and binding sites on *HOXD-AS2* promoter (**c**) were predicted by JASPAR (https://jaspar.genereg.net, accessed on 23 January 2022). (**d**) ChIP assays tested the enrichment of P1/P2/P3 fragments on the *HOXD-AS2* promoter in glioma cells LN229 and A172; anti-IgG was used as the negative control group. (**e**,**f**) ChIP assays tested the enrichment of P1/P2 fragments on the *HOXD-AS2* promoter in glioma cells, LN229 and A172 (**e**), astrocyte cell HA (**f**) and anti-IgG as the negative control group. (**g**) LN229 transfected with *TFE3* siRNAs and performed ChIP-qPCR as above. (**h**) Luciferase activity assays were performed in astrocyte cell HA-sp and glioma cells included LN229, U87MG and A172, which were transfected with pGL3-basic vector or *HOXD-AS2* promoter-containing pGL3 reporter vector and *TFE3* siRNAs; firefly luciferase activity was detected and normalized by renilla luciferase activity. The data are shown as the means ± s.d. Two-tailed Student’s *t*-test was used in (**d**–**g**). (**h**) was compared with control by ANOVA. Significant results were presented as * *p* < 0.05, ** *p* < 0.01, *** *p* < 0.001, **** *p* < 0.0001.

Based on the TCGA and GTEx database, we obtained that *TFE3* was specifically overexpressed in gliomas among different cancers (Figure 3a). In CGGA database, patients with high *TFE3* expression had shorter survival (Figure S3a). The expression of *TFE3* was lower in gliomas with 1p19q co-deletion (Figure S3b). These analyses found similar results for *TFE3* and *HOXD-AS2* (Figure 1f and Figure S1d). The expression of *TFE3* in astrocyte cell lines and glioblastoma cell lines was also detected by RT-qPCR and Western blot. According to Western blot, *TFE3* was up-regulated in glioblastoma cell lines, especially in LN229 and A172 (Figure S3c,d). However, the RT-qPCR result (Figure S3c) demonstrated that *TFE3* expression was not significantly up-regulated on the RNA level. This might be related to post-translational modification. We hypothesized that in LN229 and A172, the mRNA translation efficiency of *TFE3* was increased, leading to an increase in *TFE3* expression on the protein level. Another possibility is that the *TFE3* protein degradation was inhibited, resulting in increased accumulation of *TFE3* on the protein level. 

To verify whether *TFE3* can bind to the *HOXD-AS2* promoter, we found that *TFE3* had two motifs and six binding sites at the 2270 nucleotides upstream of *HOXD-AS2* by JASPAR. Then, we designed three pairs of primers for ChIP-qPCR. P1 represented the sequence located 1826–2021 bp upstream of *HOXD-AS2*, P2 represented the sequence located 1534–1684bp upstream of *HOXD-AS2* and P3 represented the sequence located 396–587 bp (Figure 3b,c). It was found that *TFE3* could be enriched in the region upstream of *HOXD-AS2* by ChIP-qPCR (Figure 3d), which confirmed the interaction between the *TFE3* and *HOXD-AS2* promoter. Studies have demonstrated that *TFE3* is associated with H3K27ac [40]. Based on the ChIP-seq data, we selected two pairs of primers with high peaks of H3K27ac for CHIP-qPCR. P1 represented the sequence located 1826–2021 bp upstream of *HOXD-AS2* and P2 represented the sequence located transcription start site of *HOXD-AS2* (chr2:176136890–176137010). ChIP-qPCR detected enrichment of H3K27ac upstream of *HOXD-AS2* in glioblastoma cell lines, including LN229 and A172 (Figure 3e). However, H3K27ac signal could not be detected in astrocyte cell HA by using the same primers (Figure 3f). To investigate whether *TFE3* affected the level of histone acetylation, ChIP-qPCR was performed after knocking down *TFE3* in LN229. The results demonstrated that the knockdown of *TFE3* inhibited the recruitment of H3K27ac (Figure 3g). The data above demonstrated that *HOXD-AS2* was induced by *TFE3* and H3K27ac in glioma cells. We also detected *HOXD-AS2* promoter activity after the knockdown of *TFE3* by transfecting pGL3 plasmid (Figure 2c) and detecting the relative luciferase activity. It was found that the knockdown of *TFE3* decreased the activity of the *HOXD-AS2* promoter (Figure 3h), indicating that *TFE3* could activate the transcription of *HOXD-AS2*.

### 2.4. miR-661 Degrades HOXD-AS2 in Glioblastoma Cells

We had demonstrated that *TFE3* could promote *HOXD-AS2* production at the transcriptional level. Next, we considered whether the maintenance of overexpression of *HOXD-AS2* in gliomas was related to the regulation of RNA stability.

**Figure 4:**
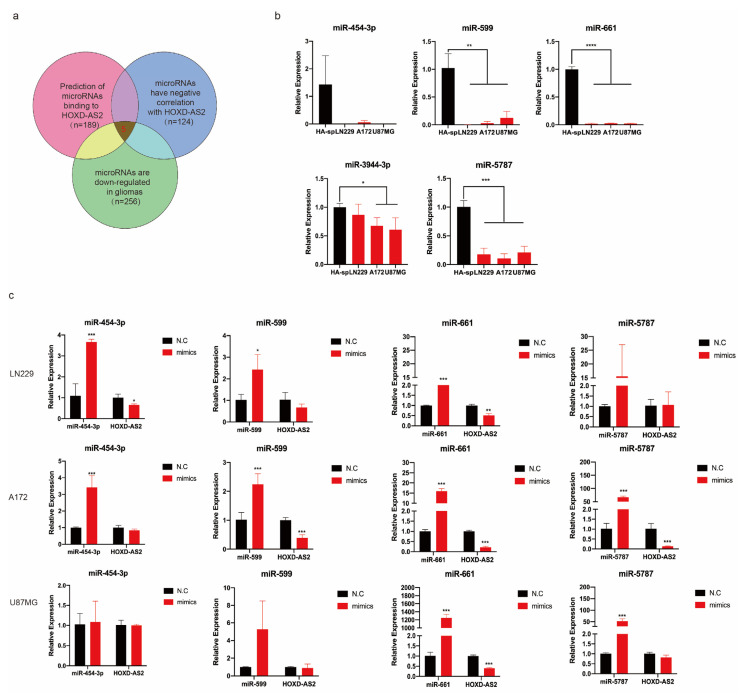

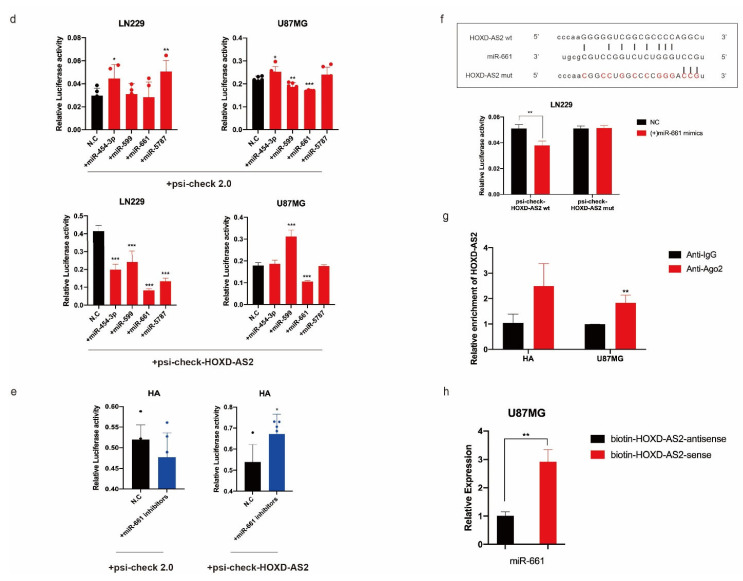
*miR-661* degraded *HOXD-AS2* at post-transcriptional level. (**a**) Flow chart provided for screening of potential miRNAs, which affected the expression of *HOXD-AS2* in gliomas. (**b**) RT-qPCR demonstrated the expression of predicted miRNAs in astrocyte and glioblastoma cell lines. (**c**) miRNAs mimics were transfected into glioblastoma cell lines LN229, A172 and U87MG to investigate the expression of *HOXD-AS2* by RT-qPCR. (**d**) Luciferase activity assays were performed in LN229 and U87MG cells, which were transfected with psi-check2.0 vector and miRNAs mimics, psi-check-*HOXD-AS2* and miRNAs mimics. Firefly luciferase activity was detected and normalized by renilla luciferase activity. (**e**) Astrocyte cell line HA was transfected with psi-check2.0 vector and *miR-661* inhibitors, psi-check-*HOXD-AS2* and *miR-661* inhibitors, then, the relative luciferase activity and firefly luciferase activity was detected, with the latter normalized by renilla luciferase activity. (**f**) Dual luciferase reporter assays were conducted with wild type and mutant type (putative binding sites for *miR-661* were mutated) luciferase reporter vectors (down). Up panel, sequence alignment of *miR-661* and its predicted binding sites in *HOXD-AS2*. (**g**) RNA immunoprecipitation with anti-Ago2 was used to assess endogenous Ago2 binding to *HOXD-AS2* in HA and U87MG cells, IgG was used as the negative control, and *HOXD-AS2* expression was detected by RT-qPCR. (**h**) Biotin-coupled RNA pull-down was used to examine the interaction of *HOXD-AS2* and miR661, and biotin-*HOXD-AS2*-antisense probe was used as the negative control. The data are shown as the means ± s.d. Two-tailed Student’s *t*-test was used in (**b**–**h**). Significant results were presented as * *p* < 0.05, ** *p* < 0.01, *** *p* < 0.001, **** *p* < 0.0001.

We previously confirmed that *HOXD-AS2* was preferentially localized in the cytoplasm (Figure 1e), so we hypothesized that microRNAs (miRNA) may degrade *HOXD-AS2* at the post-transcriptional level. Five miRNAs were selected according to the following three conditions: (1) used the full-length sequence of *HOXD-AS2* to predict miRNAs bound to *HOXD-AS2* in starBase (https://starbase.sysu.edu.cn, accessed on 23 January 2022) [44], miRDB (http://mirdb.org, accessed on 23 January 2022) [45,46] and LNCedting (http://bioinfo.life.hust.edu.cn/LNCediting/, accessed on 23 January 2022) [47]; (2) found miRNAs that were negatively correlated with *HOXD-AS2* in TCGA and CGGA cohort; (3) searched for miRNAs that were down-regulated in gliomas via miRCancer (http://mircancer.ecu.edu/search.jsp, accessed on 23 January 2022) [48,49] (Figure 4a). Supplementary Table S2 provided for all predicted miRNAs. Firstly, we examined the expression of five miRNAs in astrocyte cell HA-sp and glioblastoma cell lines, including LN229, A172 and U87MG (Figure 4b). *miR-3944-3p* was excluded according to the degree of down-regulation. Then, miRNAs mimics were transfected into three glioblastoma cell lines and detected the expression of *HOXD-AS2* by RT-qPCR. After overexpression of *miR-454-3p*, the expression of *HOXD-AS2* did not obviously change, but the rest could down-regulate *HOXD-AS2* (Figure 4c). We constructed the psi-check2.0 recombinant vector containing the full-length sequence of *HOXD-AS2* and co-transfected with miRNAs mimics to detect relative luciferase activity. We found that only *miR-661* could degrade *HOXD-AS2* in LN229 and U87MG cells (Figure 4d). In contrast, co-transfection with *miR-661* inhibitors in astrocyte cell HA led to the increase of relative luciferase activity, suggesting that *HOXD-AS2* became more stable when *miR-661* was inhibited (Figure 4e). Based on the predicted binding sequences of *miR-661* with *HOXD-AS2* from the LNCedting, we designed the mutant sequences in the full-length of *HOXD-AS2*. The wild type (wt) and mutant (mut) plasmids were respectively co-transfected with *miR-661* mimics in the LN229 cell and detected the relative luciferase activity. The results demonstrated that the mutant group did not cause the decrease of relative luciferase activity (Figure 4f). 

In order to verify whether there was an endogenous combination between *HOXD-AS2* and *miR-661*, we performed RNA immunoprecipitation (RIP) and biotin-coupled RNA-pull down technique. Ago2-RIP assays affirmed that *HOXD-AS2* was enriched in Ago2 groups; anti-IgG was used as the negative control (Figure 4g), indicating that *HOXD-AS2* existed in Ago2-related RNA-induced silencing complexes (RISCs). The RNA pull-down assay was performed to figure out whether *miR-661* could combine with *HOXD-AS2* in glioma cells. The results attested that *miR-661* was significantly harvested in *HOXD-AS2*-sense probe groups in the U87MG cell; *HOXD-AS2*-antisense probe was used as the negative control (Figure 4h). The above results indicated that *miR-661* could endogenously bind to *HOXD-AS2* in U87MG.

### 2.5. Overexpression of HOXD-AS2 Mediated by TFE3 Promotes Cell Cycle Progression

**Figure 5:**
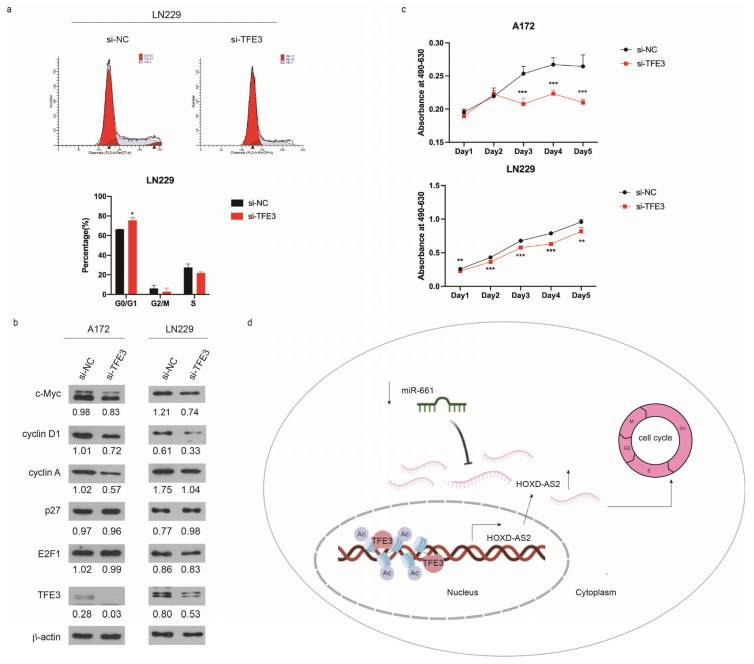
*TFE3* regulated cell cycle progression in gliomas. (**a**) Cell cycle was measured by PI staining followed by flow cytometry in LN229. Two-tailed Student’s *t*-test was used and * *p* < 0.05. (**b**) Western blot detected cell cycle-associated proteins when knocked down from *TFE3* in A172 and LN229 cells. (**c**) Growth curve of A172 cells transfecting *TFE3* siRNAs by MTS assay. Data are presented as the mean ± SEM. Two-tailed Student’s *t*-test was used at each time point. (**d**) Schematic of the proposed mechanism of *TFE3* and *miR-661* mediated *HOXD-AS2* in glioma cells. Significant results were presented as * *p* < 0.05, ** *p* < 0.01, *** *p* < 0.001.

Based on our previous work, *HOXD-AS2* could promote cell cycle in glioblastomas [17]. As expected, *TFE3* silencing dramatically induced a G1–S arrest (Figure 5a). Meanwhile, we detected the expression of cell cycle-related proteins after knockdown of *TFE3* by Western blot (Figure 5b). c-Myc stimulates proliferation in part by activating expression of CDK4 and several of the cyclins, which promote entry into S phase [50]; cyclin D1 is an essential regulator of the G1–S transition [51]; during cell-cycle progression, cyclin A level increases at the onset of S-phase and contributes to the stimulation of DNA synthesis [52]; E2F1 belongs to the E2F family of transcription factors that regulate the expression of genes involved in cell cycle progression, especially at the G1 to S phase boundary [53]; p27 is able to inhibit cyclin-dependent kinases (CDK) activity and thus participates in cell cycle regulation. The results demonstrated that the expression of c-Myc, cyclin D1, cyclin A, and E2F1 decreased after knocking down *TFE3*, suggesting that the *TFE3*-mediated overexpression of *HOXD-AS2* promoted the cell cycle progression in glioma cells. When *TFE3* was knocked down, p27 was significantly up-regulated in LN229, but almost unchanged in A172. The relationship between p27 and *TFE3* remains to be investigated. We then examined the effect of *TFE3* on cell proliferation by MTS (3-(4,5-Dimethylthiazol-2-yl)-5-(3-carboxymethoxyphenyl)-2-(4-sulfophenyl)-2H-tetrazolium) assay and found that the proliferation of glioblastoma cell lines, A172 and LN229, also decreased after knocking down *TFE3* (Figure 5c).

## 3. Discussion

Further investigations of the molecular biological characteristics of gliomas, and clinical trials to identify more potential molecular markers, contribute to comprehending the pathogenesis of gliomas. *HOXD-AS2*, as a kind of lncRNA that is specifically up-regulated in gliomas, has its own significance and value in research. We explored the mechanism of lncRNA after previously discovering its function in glioblastomas. It is worth mentioning that we did not prioritize the study of the downstream regulatory mechanism but focused on exploring the cause of the specific overexpression of *HOXD-AS2* in glioblastomas.

In this study, we found that *TFE3*, as a transcriptional regulator, could bind to the *HOXD-AS2* promoter region and recruit H3K27ac to promote *HOXD-AS2* expression. After *HOXD-AS2* was transported to the cytoplasm, *miR-661* could degrade *HOXD-AS2* through incomplete complementary binding. The expression of *miR-661* was down-regulated in glioblastomas, so the degradation process was inhibited and *HOXD-AS2* expression stayed at a high level in glioblastomas (Figure 5d). Furthermore, we found that the high expression of *TFE3* in glioma was also specific and the level of H3K27ac upstream of *HOXD-AS2* was low in astrocyte cells. The results confirmed it was specific that *TFE3* mediated *HOXD-AS2* in gliomas. There may be other pathways that co-regulate the high expression of *HOXD-AS2*, such as the previously mentioned TGF-β signaling pathway, which can activate *HOXD-AS2* in drug-resistant glioma cells [18]. In earlier years, many studies report that *TFE3* promotes the expression of genes downstream of the TGF-β signaling pathway [21,22,23,54,55]. In the future, we may be able to explore whether *TFE3* and the TGF-β signaling pathway are related with regulating *HOXD-AS2* expression in gliomas.

We had known from the previous report that *TFE3* can act as an activator to promote the accumulation of H3K27ac in the inflammatory response [40]. We did not study how *TFE3* recruited H3K27ac in gliomas, which could be a direction for future exploration. H3K27ac is associated with gene activation and is mainly enriched in promoter and enhancer regions [42]. The first step in gene transcription occurs when the chromatin changes from compact to loose, meaning that the chromatin becomes more open so that subsequent transcription factors can bind to the DNA [56,57]. Histone modification plays a key role in regulating the degree of chromatin compactness. There are many studies on histone modifications and gene regulation. Histone modifications can affect lncRNAs expression, and lncRNAs can also regulate downstream genes through histone modifications. It has been reported that H3K27ac can activate the lncRNA *CCAT1*, and *CCAT1* affects cell proliferation and migration by regulating the expression of *SPRY4* and *HOXB13* in esophageal squamous carcinoma [58]. lncRNA *GClnc1* promotes gastric carcinogenesis and progression by regulating *SOD2* through histone acetylation [59].

In 2016, the WHO central nervous system added *H3K27M* mutant diffuse midline gliomas to diffuse astrocytic and oligodendrocyte tumors [60]. *H3K27M* mutations frequently occur in high-grade gliomas (HGG) in the midline of the brain, including those in the thalamus, brainstem and spinal cord in children and adolescents [61]. *H3K27M* mutation was found in 80% of children with diffuse intrinsic pontine glioma (DIPG) [62]. In recent years, it was found that H3K27ac increased in H3K27M HGG genome, and the enrichment of H3K27ac increased the expression of repeat elements in H3K27M HGG, which provided a theoretical basis for H3K27M HGG epigenetic therapy [63].

In the past decades, miRNAs have been demonstrated to be extensively deregulated in cancers, highlighting their important role in tumor development [64]. *miR-661* is firstly found in breast cancer as a tumor suppressor [65], but *miR-661* performs various functions in a cell-specific mode. It has been reported that *miR-661* is up-regulated in non-small cell lung cancer (NSCLC) tissues and promotes cell proliferation, migration and invasion in NSCLC cells. *miR-661* directly targets Rb1, which interacts with E2F1 and mediates the EMT process in NSCLC [66].

p53 is a well-known oncogene, and it has been reported that *miR-661* targets both *mdm2* and *mdm4* mRNAs to enhance the oncogenic function of p53 [67]. The report, which shows the oncogenic effect of *miR-661* in gliomas, is consistent with our studies and further confirms the negative regulation of *HOXD-AS2* and *miR-661*. When people explored the relationship between lncRNAs and miRNAs, they often focused on ceRNA regulatory networks. However, the regulatory relationship was usually complex, and there were other regulation approaches besides ceRNA. miRNAs could also directly regulate the stability of lncRNAs. For example, *EBV-miR-BART6-3p* (*BART6*) inhibits the migratory infiltration of EBV-associated tumor cells by reversing epithelial mesenchymal transition (EMT), and BART6 directly binds to lncRNA *LOC553103* and leads to the down-regulation of *LOC553103* expression [68].

In summary, our work revealed the mechanism that *TFE3* and *miR-661* specially regulated *HOXD-AS2* in glioma overexpression, and we hope that this work will pave the way for exploring gene specific expression. The differentially expressed lncRNAs, such as *HOXD-AS2*, will also be used as new diagnostic markers for gliomas in the future.

## 4. Materials and Methods

### 4.1. Cell Culture

Astrocyte cell lines HA and HA-sp were purchased from ScienCell Research Laboratories (Carlsbad, CA, USA) and cultured with astrocyte medium (catalog 1801). The human glioblastoma cell U87MG was purchased from the American Type Culture Collection (ATCC, Manassas, VA, USA) and was maintained in MEM (modified Eagle’s medium) (Gibco, Carlsbad, CA, USA) supplemented with 1 mM sodium pyruvate and 1% (vol/vol) non-essential amino acids (NEAA), besides 10% fetal bovine serum (FBS), 100 U/mL penicillin and 100 mg/mL streptomycin. The human glioblastoma cell lines, A172 and LN229, T98G and H4, were purchased from ATCC; SNB19 was obtained from the Beijing Tiantan Hospital of Capital Medical University, Beijing, China. These human glioblastoma cell lines were cultured in DMEM (Dulbecco modified Eagle medium)/high-glucose medium (Gibco, Carlsbad, CA, USA) and supplemented with 10% FBS, 100 U/mL penicillin and 100 mg/mL streptomycin. The isolation, culture and identification of GSC2, T2-4, T12-1 and U87-SLC cells were performed, as described previously [69,70]. All cells were maintained at 37 °C in a humidified atmosphere of 5% CO_2_.

TGF-β1 was dissolved as a liquid according to the manufacturer (R&D, 100-B-001). We added TGF-β1 to LN229 and A172 containing DMEM complete medium at the concentrations of 1 ng/mL, 2 ng/mL and 4 ng/mL and treated for 48 h.

### 4.2. RNA Preparation and Quantitative Reverse Transcription PCR (RT-qPCR)

Total RNA extraction from cells was performed using the RNeasy Mini Kit (QIAGEN, Hilden, Germany). Nucleoplasmic separation was performed by the NE-PER Nuclear and Cytoplasmic Extraction Reagents kit (Thermo, Waltham, MA, USA). After obtaining cytoplasmic and nuclear fractions, the RNA was purified using Trizol reagent (Invitrogen, Carlsbad, CA, USA). The RNA was reversely transcribed into first-strand complementary DNA (cDNA) using a reverse transcription system (Trans Gene, Beijing, China). High-Capacity cDNA Reverse Transcription Kit (Applied Biosystems, Foster City, CA, USA) was used to miRNAs reverse transcription. Quantitative RT-PCR was carried out using SYBR Green (TAKARA, Otsu, Shiga, Japan). U6 and GAPDH were used as endogenous controls. The primers are shown in Supplementary Table S3.

### 4.3. Protein Extraction and Western Blot

Protein extraction and Western blot analysis were performed, as previously described [17]. Cells were lysed in TNTE buffer with protease inhibitors (Sigma Aldrich, St. Louis, MO, USA). After incubation for 30 min on ice, the homogenate was centrifuged at 12,000× *g* for 30 min. The supernatants were stored at −80 °C for a long time. The protein quantitation was performed by Pierce BCA protein assay kit (Thermo, 23227) according to the manufacturer’s protocol. The Bovine Serum Albumin Standard (BSA) was diluted to 0.5 mg/mL for the preparation of standard curves. For Western blot analysis, the protein samples were separated on a 10% SDS-PAGE gel and transferred to nitrocellulose membranes (Amersham, Sweden). The membrane was blocked with 5% skim milk in TBST for 1 h. The primary antibodies that we used are listed in Supplementary Table S4, with anti-β-actin as an internal control. The primary antibodies were revealed using the appropriate secondary antibody conjugated to peroxidase and enhanced chemiluminescence. Anti-mouse IgG (ZB-2305, ZSGB-BIO, Beijing, China) and anti-rabbit IgG (ZB-2301, ZSGB-BIO, Beijing, China) were used as secondary antibodies. The concentration of the antibodies depends on the instruction by the manufacturer. All primary antibodies were incubated overnight at 4 °C on a shaker and all secondary antibodies were incubated at room temperature for 2–3 h.

### 4.4. Plasmid Construction and Cell Transfection

INTERFERin reagent (PolyPlus, Strasbourg, France) was used to transfect small interfering RNAs (siRNA) (20 nM), RNAiMAX (Invitrogen, Carlsbad, CA, USA) was used to transfect miRNA mimics (20 nM) and miRNA inhibitors (20 nM), FuGENE6 (Promega, Madison, WI, USA) was used to transfect the plasmids into the astrocyte cell lines and glioblastoma cell lines. All siRNAs’ sequences designed for specific targets are listed in Supplementary Table S5, and purchased from Gene Pharma (Shanghai, China). We synthesized full-length complementary cDNAs of *HOXD-AS2* and cloned it into the expression vector PCIG (a gift from Professor WeiMin Zhong). miRNAs mimics and inhibitors were purchased from Sangon (Shanghai, China). The sequences are also shown in Supplementary Table S5. 

### 4.5. Flow Cytometry

Cell cycle was analyzed by flow cytometry and detected, as previously reported [71]. We used propidium iodide (PI)-stained cells. The data were analyzed by ModFit LT software.

### 4.6. Cell Proliferation Analysis

The MTS assay (Promega, Madison, WI, USA) was used to evaluate the effects of *TFE3* on the proliferation of glioma cells. Cells were seeded at a density of 2500 cells per well and 100 μL of medium per well in 96-well plates. Twenty microliters of MTS (2 mg/mL DPBS)/PMS (0.92 mg/mL DPBS) detection solution was added to each well and incubated for 2 h. Absorbance was measured with a test wavelength of 490 nm and a reference wavelength of 630 nm to obtain sample signal. 

### 4.7. Dual Luciferase Reporter Assay

To explore the transcriptional level of *HOXD-AS2* promoter, the cells were transfected with the individual reporter plasmid pGL3-*HOXD-AS2* promoter constructs (500 ng/well) and pRL-TK (50 ng/well) as an internal control; pGL3-basic was used as the control and pRL-TK (Promega, Madison, WI, USA) was used as the internal reference plasmid.

To confirm whether miRNAs could bind *HOXD-AS2*, cells were transfected with miRNA mimics or inhibitors (Sangon, Shanghai, China) (20 nM). After 20 h, the cell were transfected with the luciferase reporter vectors psi-CHECK 2.0 (Promega, Madison, WI, USA) containing the full-length sequences of *HOXD-AS2* or mutant sequences purchased from Tsingke (Beijing, China) to detect the relative luciferase activity.

After transfection of plasmids for 48 h, the luciferase activity was measured at a dual-luciferase reporter assay system (Promega, Madison, WI, USA). The experiments were performed in triplicate and the results were indicated as the means ± s.d. Firefly luciferase activity was normalized with renilla luciferase activity for each transfected well. 

### 4.8. Chromatin Immunoprecipitation (ChIP)-qPCR

Pierce™ Agarose ChIP kit (Thermo, Waltham, MA, USA) was used to identify the enrichment of *TFE3* and H3K27ac on the *HOXD-AS2* promoter. Briefly, LN229 and A172 cells were incubated with formaldehyde for 10 min to obtain the DNA-protein crosslinks. Then, cell lysates were subjected to sonicate to get the chromatin fragments and specific antibody or IgG (the negative control) were used for immunoprecipitation. Later, RT-qPCR analysis was employed to analyze the precipitated chromatin DNA. The PCR primers are listed in Supplementary Table S1.

### 4.9. DNA Pull-Down and Mass Spectrometry

*HOXD-AS2* promoter pull-down was performed by Dynabeads kilobaseBINDER kit (Thermo, Waltham, MA, USA) according to the manufacturer’s protocol. Firstly, 5′Biotin-labeled probe was synthesized by PCR and 5′Biotin-labeled primers were purchased from Sangon (Shanghai, China). The DNA-magnetic beads mixture and the nuclear protein were incubated overnight, and the nuclear protein was extracted from the NE-PER Nuclear and Cytoplasmic Extraction Reagents kit (Thermo, Waltham, MA, USA). The supernatant was obtained for further mass spectrum (Applied Protein Technology, Shanghai, China). The protein peptide samples were digested by protein endonuclease (usually Trypsin) and the samples of enzymatic hydrolysis were analyzed by nanoLC-QE LC-MS/MS. Finally, the LC-MS/MS data were analyzed using mass spectrometry matching software, such as MASCOT, to obtain qualitative identification information of the target protein peptide molecules. The identified proteins are listed in Supplementary Table S6.

### 4.10. RNA Immunoprecipitation (RIP)-qPCR

RiboCluster Profiler RIP assay Kit (MBL, Beijing, China) was used for detecting the binding between *HOXD-AS2* and Ago2. Briefly, cell lysates were incubated with proteinA/G (Thermo, 26162) beads conjugated with indicated antibody or IgG (the negative control). Followed RT-qPCR, analysis was performed to detect the enrichment of *HOXD-AS2*.

### 4.11. Biotin-Coupled RNA Pull-Down

For biotin-coupled RNA pull-down assay, Streptavidin Dyna beads (Dyna beads M-280 Streptavidin, Thermo) coated with the biotin-labeled *HOXD-AS2* probe and antisense sequences as the negative probe were incubated with cell lysis and processed according to manufacturer’s instructions. RNAs were eluted and reverse transcribed to cDNA ss in the previous method. The expression of *miR-661* was quantified by RT-qPCR. U6 was used as the housekeeping gene.

### 4.12. 5′Rapid Amplification of cDNA Ends

5′rapid amplification of cDNA ends was performed by smarter RACE 5′/3′ kit (Takara, Otsu, Shiga, Japan) according to the manufacturer’s protocol. The gene-specific primer and sequencing result are shown in Supplementary Table S1.

### 4.13. Statistical Analysis

Data are presented as mean ± SEM and were analyzed using Prism9. Overall survival (OS) was calculated using the Kaplan–Meier method. The significance of the differences between groups was estimated with Student’s *t*-test, chi-square test or one-way analysis of variance (ANOVA), as appropriate. Statistical significance was set at *p* < 0.05. The correlations between variables were analyzed with the Pearson correlation coefficient.

## Data Availability

Additional data are available upon reasonable request via e-mail.

## Supplementary Materials

The following supporting information can be downloaded at: https://www.mdpi.com/article/10.3390/ijms23052828/s1.

## References

[1] Van Meir E.G., Hadjipanayis C.G., Norden A.D., Shu H.K., Wen P.Y., Olson J.J. (2010). Exciting new advances in neuro-oncology: The avenue to a cure for malignant glioma. CA Cancer J. Clin..

[2] Malzkorn B., Reifenberger G. (2016). Practical implications of integrated glioma classification according to the World Health Organization classification of tumors of the central nervous system 2016. Curr. Opin. Oncol..

[3] Chen R., Smith-Cohn M., Cohen A.L., Colman H. (2017). Glioma Subclassifications and Their Clinical Significance. Neurotherapeutics.

[4] Mizumoto M., Yamamoto T., Ishikawa E., Matsuda M., Takano S., Ishikawa H., Okumura T., Sakurai H., Matsumura A., Tsuboi K. (2016). Proton beam therapy with concurrent chemotherapy for glioblastoma multiforme: Comparison of nimustine hydrochloride and temozolomide. J. Neurooncol..

[5] Eckel-Passow J.E., Lachance D.H., Molinaro A.M., Walsh K.M., Decker P.A., Sicotte H., Pekmezci M., Rice T., Kosel M.L., Smirnov I.V. (2015). Glioma Groups Based on 1p/19q, IDH, and TERT Promoter Mutations in Tumors. N. Engl. J. Med..

[6] Verhaak R.G., Hoadley K.A., Purdom E., Wang V., Qi Y., Wilkerson M.D., Miller C.R., Ding L., Golub T., Mesirov J.P. (2010). Integrated genomic analysis identifies clinically relevant subtypes of glioblastoma characterized by abnormalities in *PDGFRA*, *IDH1*, *EGFR*, and *NF1*. Cancer Cell.

[7] Tan A.C., Ashley D.M., López G.Y., Malinzak M., Friedman H.S., Khasraw M. (2020). Management of glioblastoma: State of the art and future directions. CA Cancer J. Clin..

[8] Hegi M.E., Diserens A.C., Gorlia T., Hamou M.F., de Tribolet N., Weller M., Kros J.M., Hainfellner J.A., Mason W., Mariani L. (2005). MGMT gene silencing and benefit from temozolomide in glioblastoma. N. Engl. J. Med..

[9] Wakimoto H., Tanaka S., Curry W.T., Loebel F., Zhao D., Tateishi K., Chen J., Klofas L.K., Lelic N., Kim J.C. (2014). Targetable signaling pathway mutations are associated with malignant phenotype in IDH-mutant gliomas. Clin. Cancer Res..

[10] Kristensen B.W., Priesterbach-Ackley L.P., Petersen J.K., Wesseling P. (2019). Molecular pathology of tumors of the central nervous system. Ann. Oncol..

[11] Mercer T.R., Dinger M.E., Mattick J.S. (2009). Long non-coding RNAs: Insights into functions. Nat. Rev. Genet..

[12] Schmitt A.M., Chang H.Y. (2016). Long Noncoding RNAs in Cancer Pathways. Cancer Cell.

[13] Kopp F., Mendell J.T. (2018). Functional Classification and Experimental Dissection of Long Noncoding RNAs. Cell.

[14] Shi J., Dong B., Cao J., Mao Y., Guan W., Peng Y., Wang S. (2017). Long non-coding RNA in glioma: Signaling pathways. Oncotarget.

[15] Gugnoni M., Ciarrocchi A. (2019). Long Noncoding RNA and Epithelial Mesenchymal Transition in Cancer. Int. J. Mol. Sci..

[16] Vecera M., Sana J., Lipina R., Smrcka M., Slaby O. (2018). Long Non-Coding RNAs in Gliomas: From Molecular Pathology to Diagnostic Biomarkers and Therapeutic Targets. Int. J. Mol. Sci..

[17] Qi Y., Wang Z., Wu F., Yin B., Jiang T., Qiang B., Yuan J., Han W., Peng X. (2018). Long noncoding RNA *HOXD-AS2* regulates cell cycle to promote glioma progression. J. Cell Biochem..

[18] Nie E., Jin X., Miao F., Yu T., Zhi T., Shi Z., Wang Y., Zhang J., Xie M., You Y. (2021). TGF-β1 modulates temozolomide resistance in glioblastoma via altered microRNA processing and elevated *MGMT*. Neuro Oncol..

[19] Zhong X., Cai Y. (2021). Long non-coding RNA (lncRNA) *HOXD-AS2* promotes glioblastoma cell proliferation, migration and invasion by regulating the miR-3681-5p/MALT1 signaling pathway. Bioengineered.

[20] Yao L., Ye P.C., Tan W., Luo Y.J., Xiang W.P., Liu Z.L., Fu Z.M., Lu F., Tang L.H., Xiao J.W. (2020). Decreased expression of the long non-coding RNA *HOXD-AS2* promotes gastric cancer progression by targeting HOXD8 and activating PI3K/Akt signaling pathway. World J. Gastrointest. Oncol..

[21] Hua X., Liu X., Ansari D.O., Lodish H.F. (1998). Synergistic cooperation of *TFE3* and smad proteins in TGF-beta-induced transcription of the plasminogen activator inhibitor-1 gene. Genes Dev..

[22] Hua X., Miller Z.A., Wu G., Shi Y., Lodish H.F. (1999). Specificity in transforming growth factor beta-induced transcription of the plasminogen activator inhibitor-1 gene: Interactions of promoter DNA, transcription factor muE3, and Smad proteins. Proc. Natl. Acad. Sci. USA.

[23] Nijman S.M.B., Hijmans E.M., Messaoudi S.E., van Dongen M.M.W., Sardet C., Bernards R. (2006). A functional genetic screen identifies *TFE3* as a gene that confers resistance to the anti-proliferative effects of the retinoblastoma protein and transforming growth factor-beta. J. Biol. Chem..

[24] Yin X., Wang B., Gan W., Zhuang W., Xiang Z., Han X., Li D. (2019). *TFE3* fusions escape from controlling of mTOR signaling pathway and accumulate in the nucleus promoting genes expression in Xp11.2 translocation renal cell carcinomas. J. Exp. Clin. Cancer Res..

[25] Dang T.T., Back S.H. (2021). Translation Inhibitors Activate Autophagy Master Regulators *TFEB* and *TFE3*. Int. J. Mol. Sci..

[26] Zehir A., Benayed R., Shah R.H., Syed A., Middha S., Kim H.R., Srinivasan P., Gao J., Chakravarty D., Devlin S.M. (2017). Mutational landscape of metastatic cancer revealed from prospective clinical sequencing of 10,000 patients. Nat. Med..

[27] Li Z., Liu Y.H., Diao H.Y., Ma J., Yao Y.L. (2015). *MiR-661* inhibits glioma cell proliferation, migration and invasion by targeting *hTERT*. Biochem. Biophys. Res. Commun..

[28] Jin T., Liu M., Liu Y., Li Y., Xu Z., He H., Liu J., Zhang Y., Ke Y. (2020). Lcn2-derived Circular RNA (hsa_circ_0088732) Inhibits Cell Apoptosis and Promotes EMT in Glioma via the *miR-661*/*RAB3D* Axis. Front. Oncol..

[29] McLendon R., Friedman A., Bigner D., Van Meir E.G., Brat D.J., Mastrogianakis G.M., Olson J.J., Mikkelsen T., Lehman N., Aldape K. (2008). Comprehensive genomic characterization defines human glioblastoma genes and core pathways. Nature.

[30] Lonsdale J., Thomas J., Salvatore M., Phillips R., Lo E., Shad S., Hasz R., Walters G., Garcia F., Young N. (2013). The Genotype-Tissue Expression (GTEx) project. Nat. Genet..

[31] Zhao Z., Zhang K.N., Wang Q., Li G., Zeng F., Zhang Y., Wu F., Chai R., Wang Z., Zhang C. (2021). Chinese Glioma Genome Atlas (CGGA): A Comprehensive Resource with Functional Genomic Data from Chinese Glioma Patients. Genom. Proteom..

[32] Kaloshi G., Benouaich-Amiel A., Diakite F., Taillibert S., Lejeune J., Laigle-Donadey F., Renard M.-A., Iraqi W., Idbaih A., Paris S. (2007). Temozolomide for low-grade gliomas: Predictive impact of 1p/19q loss on response and outcome. Neurology.

[33] Smith J.S., Tachibana I., Passe S.M., Huntley B.K., Borell T.J., Iturria N., O’Fallon J.R., Schaefer P.L., Scheithauer B.W., James C.D. (2001). *PTEN* Mutation, *EGFR* Amplification, and Outcome in Patients With Anaplastic Astrocytoma and Glioblastoma Multiforme. JNCI J. Natl. Cancer Inst..

[34] Gao J., Aksoy B.A., Dogrusoz U., Dresdner G., Gross B., Sumer S.O., Sun Y., Jacobsen A., Sinha R., Larsson E. (2013). Integrative Analysis of Complex Cancer Genomics and Clinical Profiles Using the cBioPortal. Sci. Signal..

[35] Cerami E., Gao J., Dogrusoz U., Gross B.E., Sumer S.O., Aksoy B.A., Jacobsen A., Byrne C.J., Heuer M.L., Larsson E. (2012). The cBio cancer genomics portal: An open platform for exploring multidimensional cancer genomics data. Cancer Discov..

[36] Castro-Mondragon J.A., Riudavets-Puig R., Rauluseviciute I., Berhanu Lemma R., Turchi L., Blanc-Mathieu R., Lucas J., Boddie P., Khan A., Manosalva Pérez N. (2021). JASPAR 2022: The 9th release of the open-access database of transcription factor binding profiles. Nucleic Acids Res..

[37] Zhou K.-R., Liu S., Sun W.-J., Zheng L.-L., Zhou H., Yang J.-H., Qu L.-H. (2016). ChIPBase v2.0: Decoding transcriptional regulatory networks of non-coding RNAs and protein-coding genes from ChIP-seq data. Nucleic Acids Res..

[38] Wang H., Huo X., Yang X.-R., He J., Cheng L., Wang N., Deng X., Jingyuan F., Wang N., Wang C. (2017). STAT3-mediated upregulation of lncRNA *HOXD-AS1* as a ceRNA facilitates liver cancer metastasis by regulating SOX4. Mol. Cancer.

[39] Li D., Hsu S., Purushotham D., Sears R.L., Wang T. (2019). WashU Epigenome Browser update 2019. Nucleic Acids Res..

[40] Denholtz M., Zhu Y., He Z., Lu H., Isoda T., Döhrmann S., Nizet V., Murre C. (2020). Upon microbial challenge, human neutrophils undergo rapid changes in nuclear architecture and chromatin folding to orchestrate an immediate inflammatory gene program. Genes Dev..

[41] Wang H.-Y., Long Q.-Y., Tang S.-B., Xiao Q., Gao C., Zhao Q.-Y., Li Q.-L., Ye M., Zhang L., Li L.-Y. (2019). Histone demethylase KDM3A is required for enhancer activation of hippo target genes in colorectal cancer. Nucleic Acids Res..

[42] Hnisz D., Abraham B.J., Lee T.I., Lau A., Saint-André V., Sigova A.A., Hoke H.A., Young R.A. (2013). Super-Enhancers in the Control of Cell Identity and Disease. Cell.

[43] Zhang Z., Zheng F., Yu Z., Hao J., Chen M., Yu W., Guo W., Chen Y., Huang W., Duan Z. (2017). *XRCC5* cooperates with p300 to promote cyclooxygenase-2 expression and tumor growth in colon cancers. PLoS ONE.

[44] Li J.-H., Liu S., Zhou H., Qu L.-H., Yang J.-H. (2013). starBase v2.0: Decoding miRNA-ceRNA, miRNA-ncRNA and protein–RNA interaction networks from large-scale CLIP-Seq data. Nucleic Acids Res..

[45] Chen Y., Wang X. (2020). miRDB: An online database for prediction of functional microRNA targets. Nucleic Acids Res..

[46] Liu W., Wang X. (2019). Prediction of functional microRNA targets by integrative modeling of microRNA binding and target expression data. Genome Biol..

[47] Gong J., Liu C.-J., Liu W., Xiang Y., Diao L., Guo A.-Y., Han L. (2016). LNCediting: A database for functional effects of RNA editing in lncRNAs. Nucleic Acids Res..

[48] Xie B., Ding Q., Han H., Wu D. (2013). miRCancer: A microRNA-cancer association database constructed by text mining on literature. Bioinformatics.

[49] Martin-Sanchez F., Verspoor K. (2014). Big Data in Medicine Is Driving Big Changes. Yearb. Med. Inform..

[50] Shackney S.E., Shankey T.V. (1999). Cell cycle models for molecular biology and molecular oncology: Exploring new dimensions. Cytometry.

[51] Qie S., Diehl J.A. (2016). Cyclin D1, cancer progression, and opportunities in cancer treatment. J. Mol. Med..

[52] Dai L., Liu Y., Liu J., Wen X., Xu Z., Wang Z., Sun H., Tang S., Maguire A.R., Quan J. (2013). A novel CyclinE/CyclinA-CDK Inhibitor targets p27Kip1 degradation, cell cycle progression and cell survival: Implications in cancer therapy. Cancer Lett..

[53] Denechaud P.-D., Fajas L., Giralt A. (2017). E2F1, a Novel Regulator of Metabolism. Front. Endocrinol..

[54] Hua X., Miller Z.A., Benchabane H., Wrana J.L., Lodish H.F. (2000). Synergism between Transcription Factors *TFE3* and *Smad3* in Transforming Growth Factor-β-induced Transcription of theSmad7 Gene. J. Biol. Chem..

[55] Sirard C., Kim S., Mirtsos C., Tadich P., Hoodless P.A., Itié A., Maxson R., Wrana J.L., Mak T.W. (2000). Targeted Disruption in Murine Cells Reveals Variable Requirement for Smad4 in Transforming Growth Factor β-related Signaling. J. Biol. Chem..

[56] Pradeepa M.M., Grimes G.R., Kumar Y., Olley G., Taylor G.C.A., Schneider R., Bickmore W.A. (2016). Histone H3 globular domain acetylation identifies a new class of enhancers. Nat. Genet..

[57] Rao S.S.P., Huntley M.H., Durand N.C., Stamenova E.K., Bochkov I.D., Robinson J.T., Sanborn A.L., Machol I., Omer A.D., Lander E.S. (2014). A 3D Map of the Human Genome at Kilobase Resolution Reveals Principles of Chromatin Looping. Cell.

[58] Zhang E., Han L., Yin D., He X., Hong L., Si X., Qiu M., Xu T., De W., Xu L. (2017). H3K27 acetylation activated-long non-coding RNA *CCAT1* affects cell proliferation and migration by regulating *SPRY4* and *HOXB13* expression in esophageal squamous cell carcinoma. Nucleic Acids Res..

[59] Sun T.-T., He J., Liang Q., Ren L.-L., Yan T.-T., Yu T.-C., Tang J.Y., Bao Y.-J., Hu Y., Lin Y. (2016). LncRNA *GClnc1* Promotes Gastric Carcinogenesis and May Act as a Modular Scaffold of WDR5 and KAT2A Complexes to Specify the Histone Modification Pattern. Cancer Discov..

[60] Yi S., Choi S., Kim S.H. (2018). In Reply: Impact of H3.3 K27M Mutation on Prognosis and Survival of Grade IV Spinal Cord Glioma on the Basis of New 2016 WHO Health Organization Classification of the Central Nervous System. Neurosurgery.

[61] Krug B., De Jay N., Harutyunyan A.S., Deshmukh S., Marchione D.M., Guilhamon P., Bertrand K.C., Mikael L.G., McConechy M.K., Chen C.C.L. (2019). Pervasive H3K27 Acetylation Leads to ERV Expression and a Therapeutic Vulnerability in H3K27M Gliomas. Cancer Cell.

[62] Roig-Carles D., Jackson H., Loveson K.F., Mackay A., Mather R.L., Waters E., Manzo M., Alborelli I., Golding J., Jones C. (2021). The Long Non-Coding RNA *H19* Drives the Proliferation of Diffuse Intrinsic Pontine Glioma with H3K27 Mutation. Int. J. Mol. Sci..

[63] Duchatel R.J., Jackson E.R., Alvaro F., Nixon B., Hondermarck H., Dun M.D. (2019). Signal Transduction in Diffuse Intrinsic Pontine Glioma. Proteomics.

[64] Ali Syeda Z., Langden S.S.S., Munkhzul C., Lee M., Song S.J. (2020). Regulatory Mechanism of MicroRNA Expression in Cancer. Int. J. Mol. Sci..

[65] Reddy S.D., Pakala S.B., Ohshiro K., Rayala S.K., Kumar R. (2009). MicroRNA-661, a c/EBPα Target, Inhibits Metastatic Tumor Antigen 1 and Regulates Its Functions. Cancer Res..

[66] Liu F., Cai Y., Rong X., Chen J., Zheng D., Chen L., Zhang J., Luo R., Zhao P., Ruan J. (2017). *MiR-661* promotes tumor invasion and metastasis by directly inhibiting RB1 in non small cell lung cancer. Mol. Cancer.

[67] Hoffman Y., Bublik D.R., Pilpel Y., Oren M. (2014). *miR-661* downregulates both *Mdm2* and *Mdm4* to activate p53. Cell Death Differ..

[68] He B., Li W., Wu Y., Wei F., Gong Z., Bo H., Wang Y., Li X., Xiang B., Guo C. (2016). Epstein-Barr virus-encoded *miR-BART6-3p* inhibits cancer cell metastasis and invasion by targeting long non-coding RNA *LOC**553103*. Cell Death Dis..

[69] Hu P.-S., Xia Q.-S., Wu F., Li D.-K., Qi Y.-J., Hu Y., Wei Z.-Z., Li S.-S., Tian N.-Y., Wei Q.-F. (2017). *NSPc1* promotes cancer stem cell self-renewal by repressing the synthesis of all-trans retinoic acid via targeting *RDH16* in malignant glioma. Oncogene.

[70] Xu X., Wang L., Zang Q., Li S., Li L., Wang Z., He J., Qiang B., Han W., Zhang R. (2021). Rewiring of purine metabolism in response to acidosis stress in glioma stem cells. Cell Death Dis..

[71] Shen Y., Vignali P., Wang R. (2017). Rapid Profiling Cell Cycle by Flow Cytometry Using Concurrent Staining of DNA and Mitotic Markers. Bio Protoc..

