# Correction: Qin et al. Production and Stabilization of Specific Upregulated Long Noncoding RNA HOXD-AS2 in Glioblastomas Are Mediated by TFE3 and miR-661, Respectively. *Int. J. Mol. Sci.* 2022, *23*, 2828

**DOI:** 10.3390/ijms26010263

**Published:** 2024-12-31

**Authors:** Yiming Qin, Yingjiao Qi, Xin Zhang, Zhiang Guan, Wei Han, Xiaozhong Peng

**Affiliations:** State Key Laboratory of Medical Molecular Biology, Medical Primate Research Center, Neuroscience Center, Department of Molecular Biology and Biochemistry, Institute of Basic Medical Sciences, Chinese Academy of Medical Sciences, School of Basic Medicine Peking Union Medical College, Beijing 100005, China; oranmigi@163.com (Y.Q.); ying7jiao@126.com (Y.Q.); zx18004028155@163.com (X.Z.); guanzhiang2021@163.com (Z.G.)

The authors wish to make the following correction to this paper [1]:

In the original article, there was a mistake in Figure 3a. Due to a misuse of code in drawing using R language, the wrong image was exported. The corrected Figure 3a appears below.

The original content “we obtained that TFE3 was specifically overexpressed in gliomas among different cancers (Figure 3a)” was in Section 2.3 Results, Paragraph 1.

The corrected content in Paragraph 1 was: “we also detected the expression of *TFE3* in a variety of cancers. We found that *TFE3* expression was upregulated in both GBM and LGG compared to normal tissues (Figure 3a)”.

The original content “we found that the high expression of TFE3 in glioma was also specific” was in Section 3 Discussion, Paragraph 2.

The corrected content in Paragraph 2 was: “we found that the expression of *TFE3* was high in gliomas”.

The authors apologize for any inconvenience caused and state that the scientific conclusions are unaffected. This correction was approved by the Academic Editor. The original publication has also been updated.

## Figures and Tables

**Figure 3:**
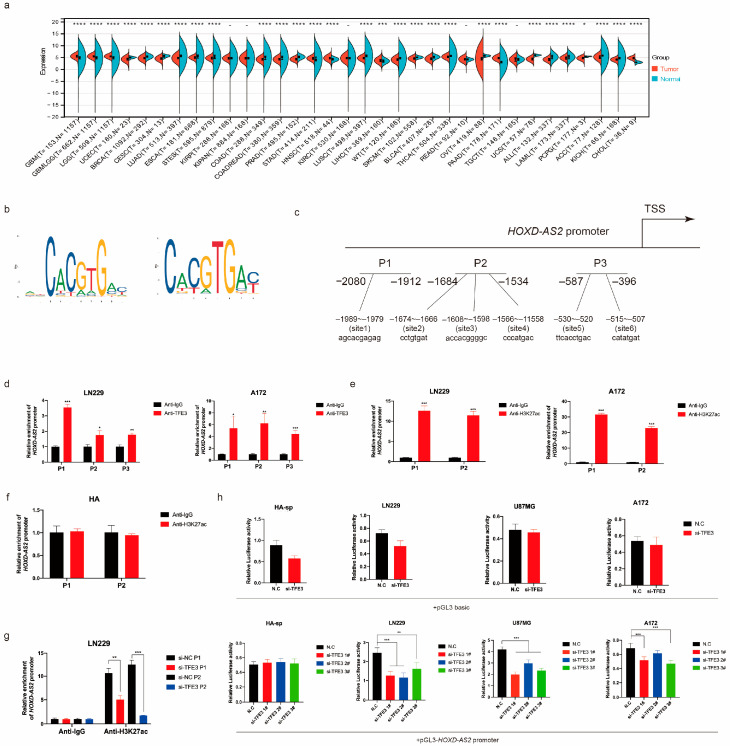
*TFE3* regulated the expression of *HOXD-AS2*. (**a**) TCGA and GTEx database demonstrated the expression of *TFE3* in different tumors. The *p*-values were calculated using a Wilcoxon test by R (version 4.0.5). Significant results were presented as * *p* < 0.05, ** *p* < 0.01, *** *p* < 0.001, **** *p* < 0.0001. (**b**,**c**) The binding motifs of *TFE3* (**b**) and binding sites on *HOXD-AS2* promoter (**c**) were predicted by JASPAR (https://jaspar.genereg.net (accessed on 23 January 2022)). (**d**) ChIP assays tested the enrichment of P1/P2/P3 fragments on the *HOXD-AS2* promoter in glioma cells LN229 and A172; anti-IgG was used as the negative control group. (**e**,**f**) ChIP assays tested the enrichment of P1/P2 fragments on the *HOXD-AS2* promoter in glioma cells LN229 and A172 (**e**), astrocyte cell HA (**f**) and anti-IgG as the negative control group. (**g**) LN229 transfected with *TFE3* siRNAs and performed ChIP-qPCR as above. (**h**) Luciferase activity assays were performed in astrocyte cell HA-sp and glioma cells, including LN229, U87MG and A172, which were transfected with pGL3-basic vector or *HOXD-AS2* promoter-containing pGL3 reporter vector and *TFE3* siRNAs; firefly luciferase activity was detected and normalized by renilla luciferase activity. The data are shown as the means ± s.d. Two-tailed Student’s *t*-test was used in (**d**–**g**). (**h**) was compared with control by ANOVA. Significant results were presented as * *p* < 0.05, ** *p* < 0.01, *** *p* < 0.001, **** *p* < 0.0001.

## References

[1] Qin Y., Qi Y., Zhang X., Guan Z., Han W., Peng X. (2022). Production and Stabilization of Specific Upregulated Long Noncoding RNA HOXD-AS2 in Glioblastomas Are Mediated by TFE3 and miR-661, Respectively. Int. J. Mol. Sci..

