# Supplementary material for: Production and Stabilization of Specific Upregulated Long Noncoding RNA HOXD-AS2 in Glioblastomas Are Mediated by TFE3 and miR-661, Respectively
Source: Int J Mol Sci. 2022 Mar 4;23(5):2828. doi: 10.3390/ijms23052828 (PMC8911140; doi:10.3390/ijms23052828)
Supplement: Supplementary file 1 [file ijms-23-02828-s001.zip › Supplementary Materials.pdf]

Supplementary Materials: Production and stabilization of specific upregulated long noncoding RNA HOXD-AS2 in glioblastomas are mediated by TFE3 and miR-661, respectively

YiMing Qin [1], YingJiao Qi[1], Xin Zhang [1], ZhiAng Guan [1], Wei Han [1,*], and XiaoZhong Peng [1,2,*]

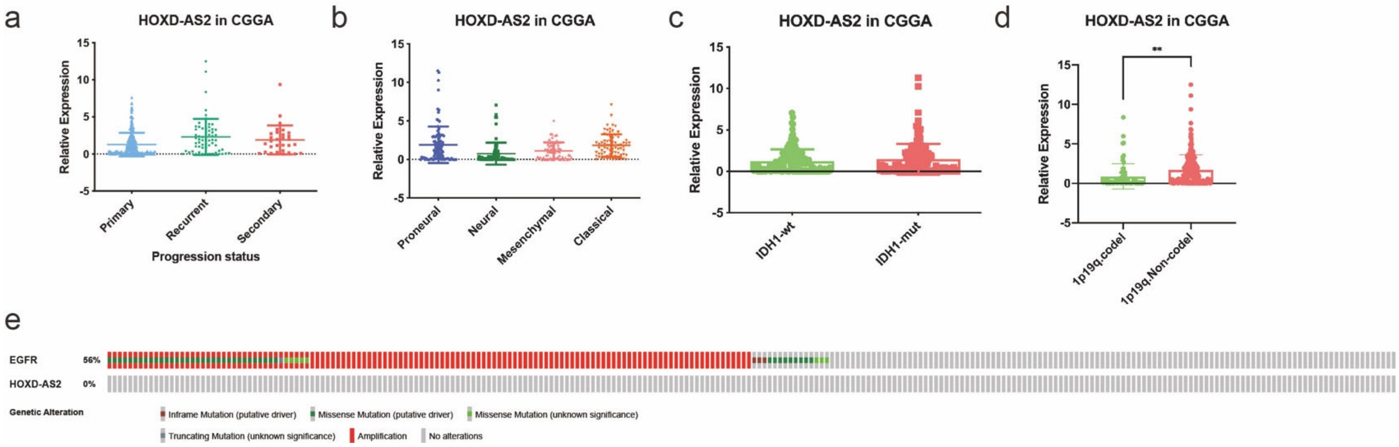

Figure S1. Analysis of *HOXD-AS2* in CGGA and cBioportal database .(**a-d**) RNA-seq data (n=325) in CGGA showed the experssion of *HOXD-AS2* in different progression statue (a) and subtypes (b), IDH mutation status (c), 1p/19q co–deletion status (d). (**e**) The CNVS section in cBioportal (https://www.cbioportal.org/, accessed on 17 February 2022). Segmented copy-number data for the selected 248 samples. *EGFR* was used as a positive control. Two-tailed Student's t test was used in (a-d). Significant results were presented as $**P<0.01$.

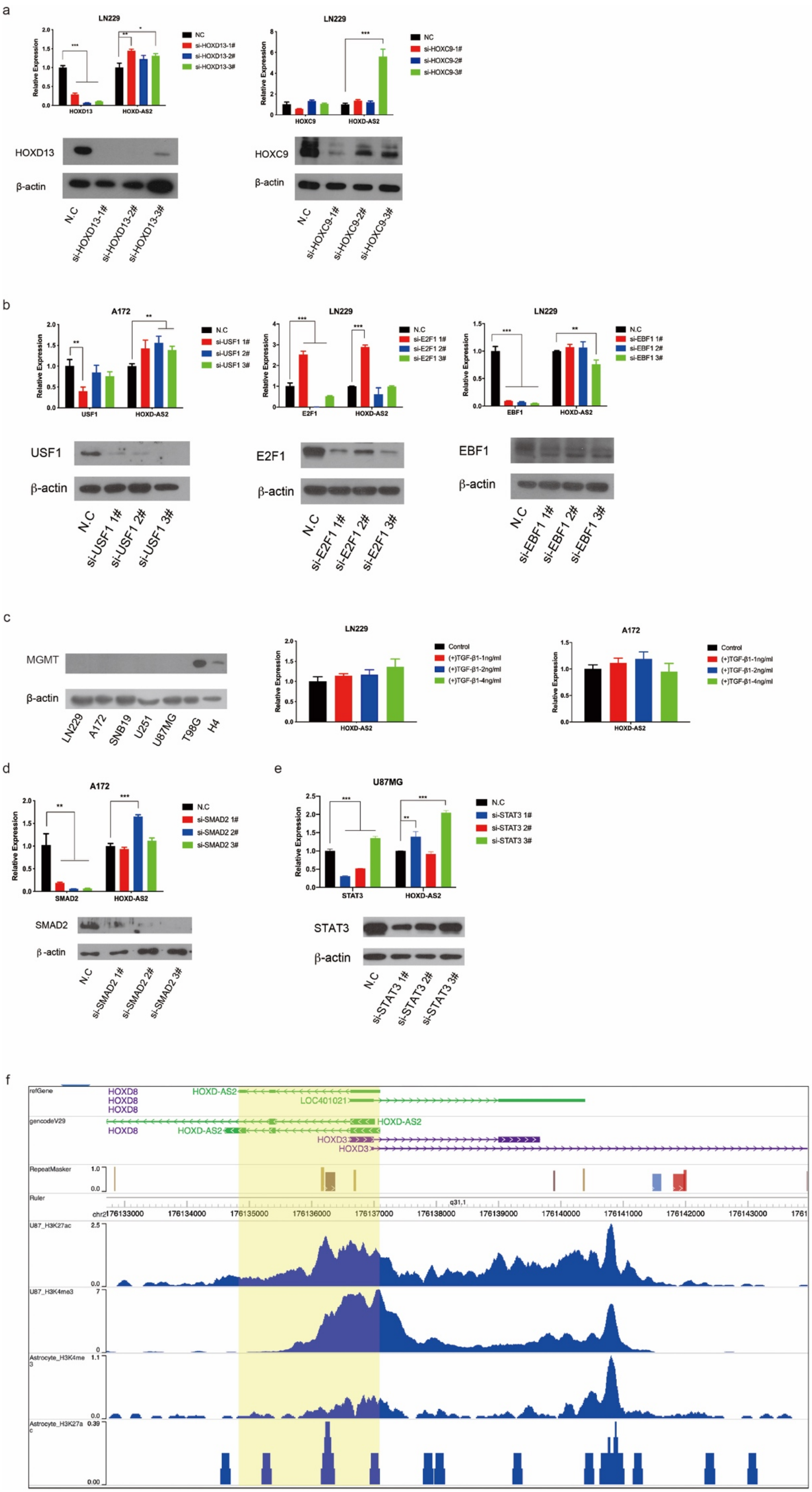
a
LN229
Relative Expression
HOXD13
HOXD-AS2
NC
si-HOXD13-1#
si-HOXD13-2#
si-HOXD13-3#
HOXC9
si-HOXC9-1#
si-HOXC9-2#
si-HOXC9-3#
β-actin
N.C
b
A172
USF1
N.C
si-USF1 1#
si-USF1 2#
si-USF1 3#
E2F1
si-E2F1 1#
si-E2F1 2#
si-E2F1 3#
EBF1
si-EBF1 1#
si-EBF1 2#
si-EBF1 3#
c
MGMT
LN229
A172
SNB19
U251
U87MG
T98G
H4
Control
(+)TGF-β1-1ng/ml
(+)TGF-β1-2ng/ml
(+)TGF-β1-4ng/ml
d
SMAD2
si-SMAD2 1#
si-SMAD2 2#
si-SMAD2 3#
e
U87MG
STAT3
si-STAT3 1#
si-STAT3 2#
si-STAT3 3#
f
refGene
HOXD8
HOXD-AS2
LOC401021
gencodeV29
HOXD3
RepeatMasker
Ruler
q31.1
chr2
176133000
176134000
176135000
176136000
176137000
176138000
176139000
176140000
176141000
176142000
176143000
U87_H3K27ac
U87_H3K4me3
Astrocyte_H3K4me3
Astrocyte_H3K27ac

Figure S2. Predicted factors which regulating *HOXD-AS2* at the transcriptional level. (**a-b**) To screen and verify whether transcription factors (*HOXD13, HOXC9, USF1, E2F1, EBF1*) regulate *HOXD-AS2* expression, the expression of *HOXD-AS2* was detected by RT-qPCR and western blot after siRNAs transfection. (**c**) The expression of *MGMT* was detected by western blot in glioblastoma cell lines (left). TGF-β1 treated glioblastoma cell lines LN229 and A172, RT-qPCR detected the expression of *HOXD-AS2* (middle and right). (**d-e**) The expression of HOXD-AS2 was detected by RT-qPCR and western blot after transfecting *SMAD2* (d) and *STAT3* (e) siRNAs. (**f**) The enrichment of H3K27ac and H3K4me3 at the *HOXD-AS2* promoter in U87MG and astrocyte cells was analyzed by Cistrome database (http://cistrome.org/db/#/). Data are presented as the mean ± SEM from three independent experiments. (a-e) were analyzed by ANOVA. Significant results were presented as *P<0.05, **P<0.01, ***P<0.001.

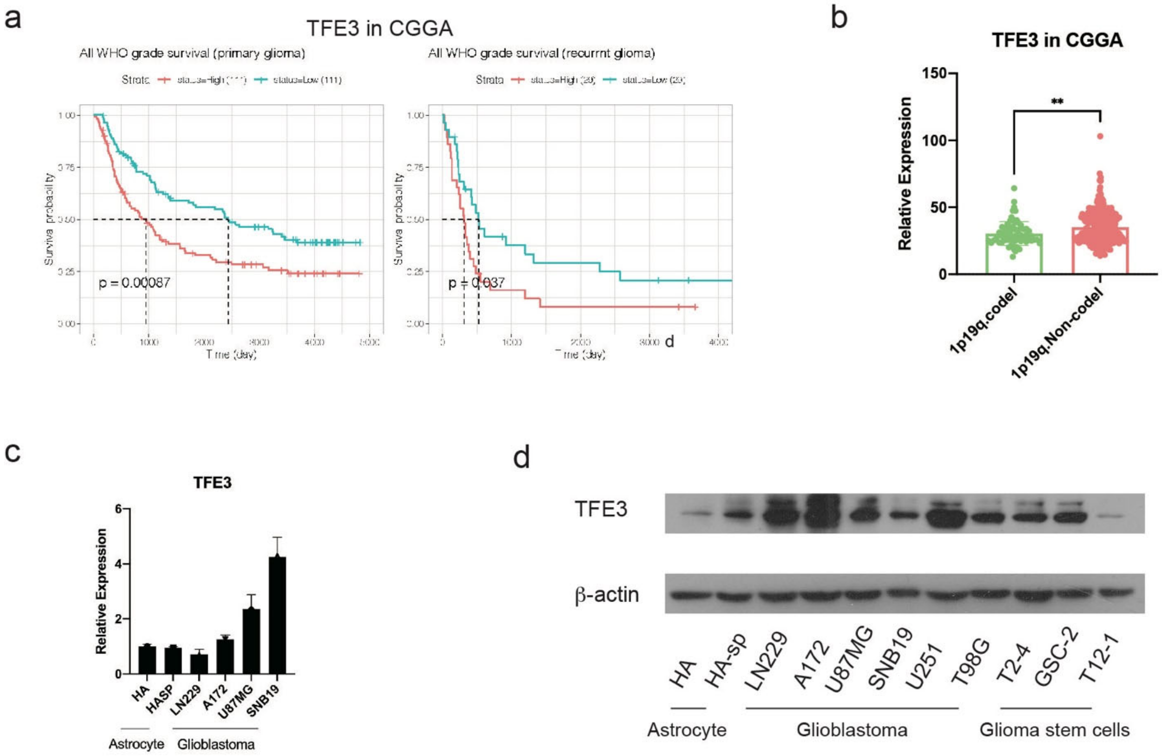

Figure S3. Analysis of *TFE3* in CGGA cohort and cell lines. (**a**) Kaplan- Meier survival analysis of OS in GBM patients based on *TFE3* expression in CGGA. (Mantel-Cox test). (**b**) The expression of *TFE3* in 1p/19q co−deletion and 1p/19q non-co−deletion in CGGA database. (**c**) *TFE3* expression in astrocyte cell lines and glioblastoma cell lines by RT-qPCR. (**d**) *TFE3* expression in astrocyte cell lines, glioblastoma cell lines and glioma stem cell lines by western blot. Two-tailed Student's t test was used in (a-d). Significant results were presented as ***P*<0.01.

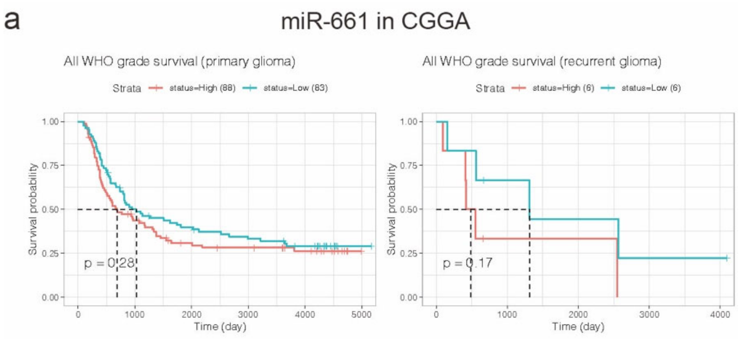

Figure S4. Analysis of *miR-661* in CGGA. **(a)** Kaplan–Meier analysis of the association between *miR-661* high or low expression and the overall survival of gliomas patients in CGGA cohort (Mantel-Cox test).
